# Interleukin‐37 promotes DMBA/TPA skin cancer through SIGIRR‐mediated inhibition of glycolysis in CD103^+^DC cells

**DOI:** 10.1002/mco2.229

**Published:** 2023-03-05

**Authors:** Fan‐lian Zeng, Xiao‐yan Wang, Ya‐wen Hu, Zhen Wang, Ya Li, Jing Hu, Jia‐dong Yu, Pei Zhou, Xiu Teng, Hong Zhou, Hua‐ping Zheng, Fu‐lei Zhao, Lin‐na Gu, Cheng‐cheng Yue, Shu‐wen Chen, Juan Cheng, Yan Hao, Qi‐xiang Zhao, Chen Zhang, Song Zou, Zhong‐lan Hu, Xiao‐qiong Wei, Xiao Liu, Guo‐lin Li, Nong‐yu Huang, Wen‐ling Wu, Yi‐fan Zhou, Wei Li, Kaijun Cui, Jiong Li

**Affiliations:** ^1^ State Key Laboratory of Biotherapy and Cancer Center West China Hospital West China Medical School Sichuan University and Collaborative Innovation Center for Biotherapy Chengdu China; ^2^ Department of Liver Surgery and Liver Transplantation West China Hospital Sichuan University and Collaborative Innovation Center of Biotherapy Chengdu China; ^3^ Laboratory of Liver Surgery West China Hospital Sichuan University Chengdu China; ^4^ Laboratory of Human Disease and Immunotherapies West China Hospital Sichuan University Chengdu China; ^5^ Department of Cardiology West China Hospital Sichuan University Chengdu China; ^6^ Department of Dermatovenereology West China Hospital Sichuan University Chengdu China

**Keywords:** CD103^+^DCs, glycolysis, interleukin‐37, SIGIRR, skin cancer

## Abstract

Interleukin 37 (IL‐37), a member of the IL‐1 family, is considered a suppressor of innate and adaptive immunity and, hence is a regulator of tumor immunity. However, the specific molecular mechanism and role of IL‐37 in skin cancer remain unclear. Here, we report that IL‐37b‐transgenic mice (IL‐37tg) treated with the carcinogenic 7,12‐dimethylbenzoanthracene (DMBA)/12‐o‐tetradecylphorbol‐13‐acetate (TPA) exhibited enhanced skin cancer and increased tumor burden in the skin by inhibiting the function of CD103^+^ dendritic cells (DCs). Notably, IL‐37 induced rapid phosphorylation of adenosine 5‘‐monophosphate (AMP)‐activated protein kinase (AMPK), and via single immunoglobulin IL‐1‐related receptor (SIGIRR), inhibited the long‐term Akt activation. Specifically, by affecting the SIGIRR‐AMPK‐Akt signaling axis, which is related to the regulation of glycolysis in CD103^+^DCs, IL‐37 inhibited their anti‐tumor function. Our results show that a marked correlation between the CD103^+^DC signature (IRF8, FMS‐like tyrosine kinase 3 ligand, CLEC9A, CLNK, XCR1, BATF3, and ZBTB46) and chemokines C‐X‐C motif chemokine ligand 9, CXCL10, and CD8A in a mouse model with DMBA/TPA‐induced skin cancer. In a word, our results highlight that IL‐37 as an inhibitor of tumor immune surveillance through modulating CD103^+^DCs and establishing an important link between metabolism and immunity as a therapeutic target for skin cancer.

## Graphical abstract

In the 7,12‐dimethylbenzoanthracene/tetradecylphorbol‐13‐acetate two‐stage skin cancer induction model, interleukin weakened the expression of CD40/CCR7 and the secretion of C‐X‐C motif chemokine ligand 9/CXCL10 in CD103^+^DCs through single immunoglobulin interleukin‐1‐related receptor‐activated protein kinase‐Akt signal axis, which leads to the suppression the function of CD8^+^T cells and accelerating the growth of tumor.

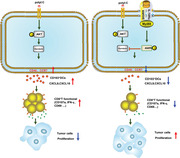

## 1 INTRODUCTION

Skin cancer is one of the most common cancers in western countries, and its global incidence is also rising rapidly.^1^, ^2^ Non‐melanoma skin cancer accounts for approximately 2–3 million cases worldwide each year and its incidence has risen rapidly in the past decades.^3^ The two‐stage cutaneous carcinogenesis induced by 7,12‐dimethylbenzoanthracene (DMBA)/12‐o‐tetradecylphorbol‐13‐acetate (TPA) is a widely accepted model for studying skin cancer and has significant homology with human squamous cell carcinoma (SCC).^4^ Dendritic cell (DC) networks play an important role in the skin's immune system. It regulates innate and adaptive immunity and participates in maintaining the stability of the tissue immune microenvironment.^5^ During tumorigenesis, skin DCs capture and present exogenous antigens and, migrate to skin‐draining lymph node (SDLN) after maturation, where they trigger an immune response by activating naive T cells.^6^, ^7^ Tissue‐resident DC subsets are widely classified as CD103^+^DC (cDC1) and CD11b^+^DC (cDC2). The development of the CD103^+^DC subgroup is regulated by the transcription factor IRF8 and cytokine FMS‐like tyrosine kinase 3 ligand (Flt3L), which promote the secretion of chemokines C‐X‐C motif chemokine ligand 9 (CXCL9) and CXCL10,^8^ recruit and induce cross‐priming of anti‐tumor CD8^+^ T cells and play a unique role in promoting anti‐tumor immunity.^9^, ^10^, ^11^, ^12^, ^13^

The IL‐1 cytokines family has been widely studied because of its regulatory function toward the immune system. Members of this family play important roles in the recruitment, activation, proliferation, and differentiation of immune cells.^14^, ^15^, ^16^, ^17^ Interleukin‐37 (IL‐37), is well‐known as an immunosuppressive molecule.^18^, ^19^ Previous studies have shown that IL‐37 relies primarily on IL‐37‐single immunoglobulin IL‐1‐related receptor (SIGIRR)‐IL‐18Rα Three‐binding combination to perform its functions.^20^ Our previous study found that in the azoxymethane/dextran sodium sulfate‐induced colorectal cancer (CRC) mouse model, IL‐37 restricted the activity of CD8^+^ T cells induced by IL‐18 through the SIGIRR.^21^ Moreover, IL‐37 affected the ability of DCs to sensitize immature and antigen‐specific T cells in the dinitrofluorobenzene (DNFB)‐induced allergic contact dermatitis.^22^ However, the immune effects and mechanism of IL‐37 in the skin cancer microenvironment remain unclear, as well as the role and mechanism of IL‐37 in DC tumor immunosurveillance. Although a murine homolog of IL‐37 has not been identified, human IL‐37 can efficiently act on murine immune cells, such as monocytes and lymphocytes, by inhibiting innate immunity and the release of proinflammatory cytokines and chemokines. Therefore, to perform our investigation we generated an IL‐37 transgenic (IL‐37tg) mouse model.^23^

Many existing studies show that have shown that cell metabolism is an important determinant of immune cell function.^24^, ^25^, ^26^, ^27^ DC expresses multiple pattern recognition receptors to activate from an immature to a mature state.^28^ DC maintains early activation by increasing glycolytic metabolism. The inhibitory of glycolysis can damage the maintenance of DCs maturation, DC movement, and C‐C chemokine receptor type 7 (CCR7) oligomerization, which is switched by the PI3K/Akt signaling pathway and is also restricted by adenosine 5‘‐monophosphate (AMP)‐activated protein kinase (AMPK).^29^, ^30^, ^31^, ^32^ IL‐37 plays a complex role in cell metabolism, including activating AMPK, inhibiting mTOR, and an increase in oxidative phosphorylation.^33^, ^34^

In our study, we investigated the function of IL‐37 on the immune microenvironment in DMBA/TPA‐induced mouse skin SCC. We showed that IL‐37 weakens the function of CD103^+^DCs and affects the activation of CD8^+^ T cells to promote SCC development. Moreover, IL‐37 inhibited the metabolism of CD103^+^DCs for a long time through the SIGIRR‐AMPK‐Akt signaling pathway. Our data confirmed the breakthrough discovery of IL‐37 in the progress of skin cancer.

## 2 RESULTS

### 2.1 Transgenic expression of IL‐37 promotes DMBA/TPA‐induced skin cancer

**FIGURE 1:**
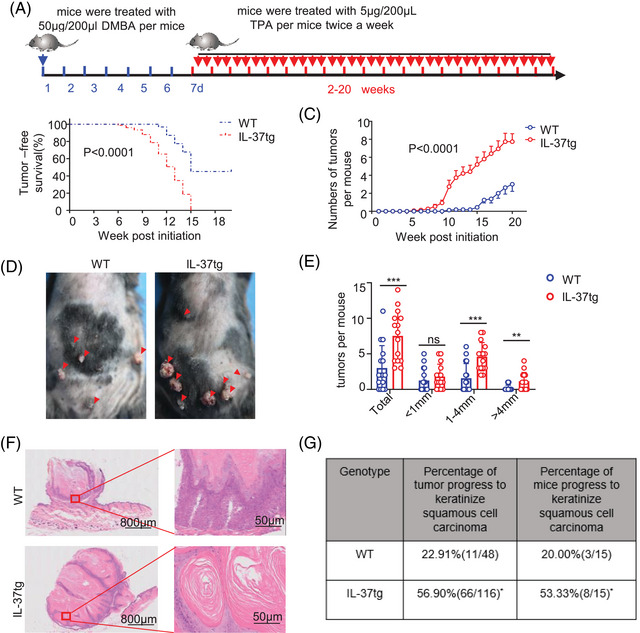
Enhanced skin carcinogenesis in interleukin‐37b‐transgenic (IL‐37tg) mice. (A) Details of the 7,12‐dimethylbenz(a)anthracene (DMBA)/12‐O‐tetradecanoylphorbol‐13‐acetate (TPA) treatment used for inducing skin cancer. Male mice aged 6–8 weeks were treated with 50 μg DMBA in 200 μl acetone one week, followed by 5 μg TPA in 200 μl acetone twice a week. (B) Tumor‐bearing ratio of IL‐37tg mice (*n* = 15/group), and wild‐type (WT) littermates (*n* = 15/group) treated with DMBA/TPA. (C) The number of tumors per mouse in IL‐37tg significantly increased compared with the WT littermates after DMBA/TPA treatment. (D) Representative macroscopic image in the skin at 20 weeks after DMBA/TPA administration, tumor development of the skin was determined. (E) Quantification of the number of skin tumors at 20 weeks after DMBA/TPA administration. (F) Representative hematoxylin and eosin (H&E) images of the tumor at 32 weeks after DMBA/TPA administration. (G) The rate of keratinized squamous cell carcinoma (SCC) tumors after DMBA/TPA treated with 20 weeks of WT and IL‐37tg mice. Data are presented as the mean  ±  SD *p  <  0.05; **p  <  0.01; ***p  <  0.001. ns, no significance, as determined by a two‐tailed Student's *t*‐test.

To investigate the role of IL‐37 in skin cancer, IL‐37tg mice were used. Compared with wild‐type (WT) mice,^21^ no difference was observed in the skin and thickness of the epidermis in WT and IL‐37tg mice (Figure S1A,B). Quantitative polymerase chain reaction (qPCR) and western blotting (WB) results displayed that the *IL‐37* gene was stably expressed in the skin of IL‐37tg mice at rest state, and also in bone marrow (BM), spleen tissue, and peripheral blood mononuclear cells (Figure S1C,D). These results showed that IL‐37 transgene does not affect the skin and expresses stably at the basal state. The skin carcinogenesis protocol was initiated with a single treatment with 50 μg DMBA followed by 5 μg TPA twice weekly (Figure 1A). IL‐37 showed a significant pro‐tumorigenic effect in mice, owing to the appearance of early tumors (Figure 1B,C). Moreover, IL‐37tg mice displayed an increased number of tumors (Figure 1D) and specifically, more tumors with a diameter greater than 1 mm compared with WT littermates (Figure 1E). Histological analysis showed that keratinizing beads were formed in the tumors of IL‐37tg mice, accompanied by more severe pathological injury (Figure 1F). These results suggested that IL‐37tg mice are more sensitive to DMBA/TPA‐induced skin cancer. Moreover, the percentage of tumors that progressed to keratinized SCC increased in IL‐37tg more than those in WT mice. The percentage of IL‐37tg mice with keratinized SCC was higher than WT mice as well (Figure 1G). These results reflected that IL‐37 promoted the growth, and deterioration of tumors.

### 2.2 Reduction in CD103^+^DCs in IL‐37tg mice during DMBA/TPA‐induced tumorigenesis

**FIGURE 2:**
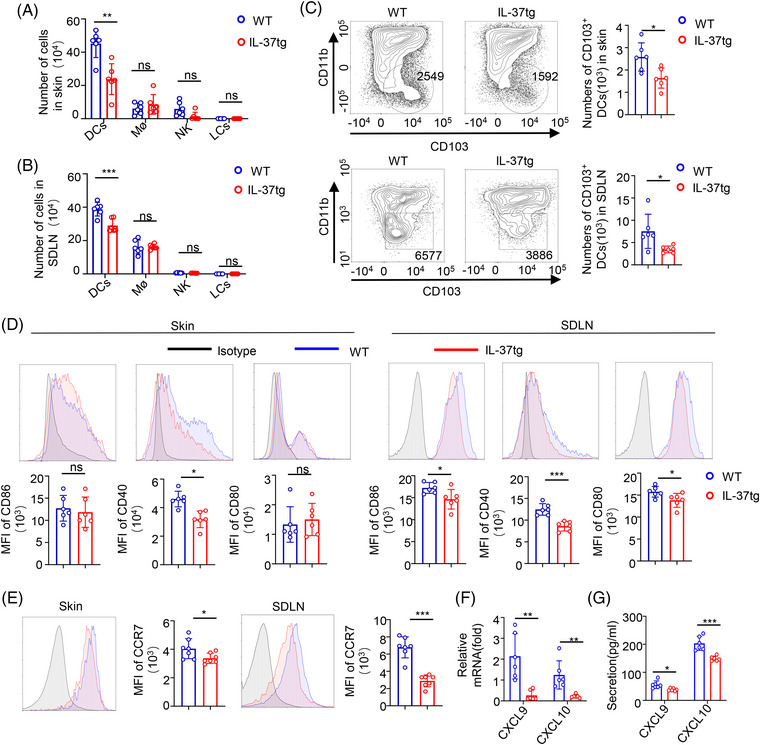
Interleukin‐37b‐transgenic (IL‐37tg) mice infiltrated fewer CD103^+^DCs than wild‐type (WT) littermates. The numbers, levels of co‐stimulator molecules, and migratory ability of CD103^+^DCs of the tumor‐bearing skin tissue and skin‐draining lymph node (SDLN) from IL‐37tg and WT mice were examined using flow cytometry after 7,12‐dimethylbenz(a)anthracene (DMBA)/12‐O‐tetradecanoylphorbol‐13‐acetate (TPA) treatment for 32 weeks. (A, B) The numbers of CD11C^+^MHC II^+^ cells (dendritic cells [DCs]), CD11B^+^F4/80^+^ cells (Mø cells), LCs(Langerhans cells), and NK1.1^+^ cells (natural killer [NK] cells) of the (A) tumor‐bearing skin tissue and (B) SDLN after IL‐37tg and WT mice, *n*  =  6/group. (C) Representative flow cytometry diagram of CD103^+^DCs (CD11C^+^CD103^+^CD11b^−^ cells) in IL‐37tg mice and WT littermates, and numbers of CD103^+^DCs in tumor‐bearing skin tissue (Upper) and SDLN (Lower), *n*  =  6/group. (D) Representative mean fluorescence intensity (MFI) (Upper) and histogram (Lower) of CD40, CD80, and CD86 (E) and CCR7 of CD103^+^DCs of tumor‐bearing skin tissue and SDLN from IL‐37tg mice and WT littermates, *n*  =  6/group. (F) C‐X‐C motif chemokine ligand 9 (CXCL9) and CXCL10 levels in the SDLN of IL‐37tg and WT mice were detected by qPCR after DMBA/TPA treatment for 32 weeks (*n* = 6/group). (G) CXCL9 and CXCL10 levels in the serum of IL‐37tg and WT mice detected by enzyme‐linked immunosorbent assay (ELISA) after treatment with DMBA/TPA for 32 weeks (*n* = 6/group). Data are presented as the mean  ±  SD. *p  <  0.05; **p  <  0.01; ***p  <  0.001. ns, no significance, as determined by a two‐tailed Student's *t*‐test.

Recent studies have shown that the main antigen‐presenting cells (APCs) in the skin contain Langerhans cells (LCs) and DCs, while the main cell populations in the skin include LCs, Dermal DC, plasmacytoid DCs (pDCs), macrophages (Mø), natural killer (NK) cells, mast cells, CD4^+^T cells, CD8^+^T cells, and γδ T cells.^35^, ^36^, ^37^ In order to have a better understanding of the role of IL‐37 in promoting tumor immunity, we evaluated the immune cells with antigen phagocytic composition of tumor‐bearing skin tissue and SDLN. No difference in the number of DC, LCs, Mø, and NK cells in the resting state was detected (Figure S2A,B). After DMBA/TPA treatment for 32 weeks, NK cells, LCs, and Mø also showed no differences between the IL‐37tg mice and their WT littermates, whereas a significant increase in DCs was observed in both the skin and SDLN of WT littermate mice than that of that detected in IL‐37tg mice (Figure 2A,B). Then, we detected the DC subgroup according to the expression of CD11b and CD103. CD103^+^DCs are extremely sparse yet remarkably capable stimulators of cytotoxic T lymphocytes (CTL). A remarkably lower number of CD103^+^DCs was observed in the skin and SDLN of DMBA/TPA‐treated IL‐37tg mice than that of WT mice, whereas no difference in CD11b^+^DCs was observed (Figure 2C, and Figure S2C), which was similar to the results obtained in the inflammation stage of skin cancer model after DMBA/TPA treatment for 6 weeks (Figure S2F–H). And we also analyzed the RNA of the pDC marker (B220, BST2, and Siglec‐H)^38^ in the DMBA/TPA SCC model of WT and IL‐37tg mice, and the results showed no significant difference (Figure S2D,E).

To clear and definite whether IL‐37 expression also impairs the maturation of CD103^+^DCs in vivo, we evaluated CD80, CD86, and CD40 expression in skin and SDLN CD103^+^DCs after DMBA/TPA treatment. DMBA/TPA treatment reduced CD80, CD86, and CD40 expression in CD103^+^DCs from IL‐37tg SDLN, however, the changes of CD80 and CD86 were not detected in CD103^+^DC from IL‐37tg skin (Figure 2D). After antigen uptake, DC increases the expression of chemokine receptor CCR7 and migrates to lymph nodes.^39^ Therefore, we analyzed CCR7 expression in CD103^+^DCs and found that the mean fluorescence intensity (MFI) of CCR7 in CD103^+^DCs was reduced in IL‐37tg mice compared with those in WT littermates (Figure 2E). CXCL9/10 is the main source of T cell‐recruiting secreted by CD103^+^DCs,^8^ and qPCR showed that CXCL9/10 messenger RNA (mRNA) levels of SDLN in WT littermates were higher than those in IL‐37tg mice (Figure 2F). No significant difference was detected in the skin (data not shown). Moreover, we examined CXCL9 and CXCL10 expression in the serum of the DMBA/TPA mice. Lower serum CXCL9 and CXCL10 levels were observed in IL‐37tg mice (Figure 2G). IL‐10, IL‐12, and IL‐2 have recently been found to be important for DCs or effector CD8 T cell‐mediated anti‐tumor immunity.^40^, ^41^, ^42^ Expression analysis through qPCR showed an increase of IL‐10 and IL‐2 mRNA levels in skin and SDLN in WT littermates compared with those in IL‐37tg mice, whereas no difference in IL‐12 was detected (Figure S2I). These results suggested that IL‐37 plays a significant role in the development and migration of CD103^+^DCs in DMBA/TPA skin cancers.

### 2.3 IL‐37 decreases the activation of anti‐tumoral CD8^+^ T cells in DMBA/TPA‐induced tumorigenesis

**FIGURE 3:**
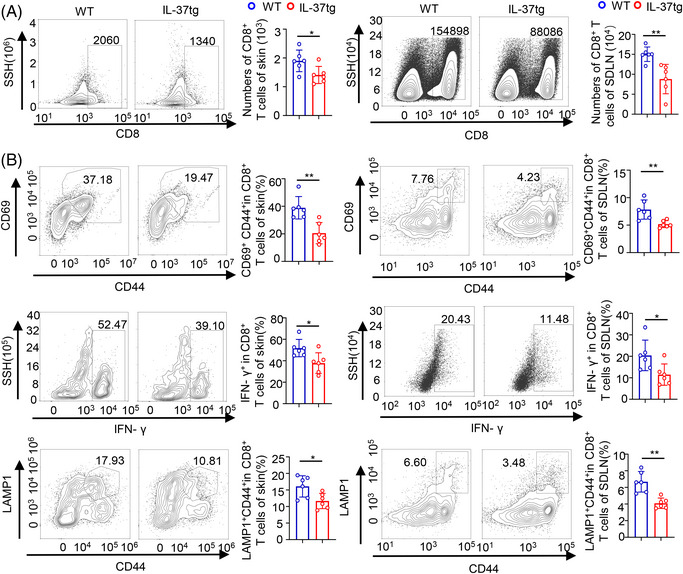
Interleukin‐37b‐transgenic (IL‐37tg) mice showed fewer CD8^+^ tumor‐infiltrating lymphocytes (TILs) than wild‐type (WT) littermates. IL‐37tg mice and WT littermates were treated with 7,12‐dimethylbenz(a)anthracene (DMBA)/12‐O‐tetradecanoylphorbol‐13‐acetate (TPA) for 20 weeks, and the numbers and abilities of CD8^+^T cells were analyzed by flow cytometry of tumor‐bearing skin tissue and skin‐draining lymph node (SDLN) from IL‐37tg mice and WT littermates after treatment with DMBA/TPA for 20 weeks. (A) Representative diagram of flow cytometry of CD8^+^ TILs (CD3^+^CD8^+^ cells) in IL‐37tg and WT mice, the number of CD8^+^ TILs cells in tumor‐bearing skin tissue (Left) and SDLN (Right), n  =  6/group. (B) Representative flow cytometric analysis of CD8^+^ TILs expressing activation markers (CD44 and CD69) and effector molecules (IFN‐γ and lysosomal‐associated membrane protein‐1 [LAMP1]) of the tumor‐bearing skin tissue and SDLN (Left), and the ratio of CD44, CD69, IFN‐γ and LAMP1 levels among CD8^+^ TILs (Right), *n*  =  6/group. Data are presented as mean  ±  SD. *p  <  0.05; **p  <  0.01; ns, no significance, as determined by a two‐tailed Student's *t*‐test.

Upon antigen uptake, CD103^+^DCs possess the unique ability of antigen cross‐presentation and activate naive CD8^+^T cells,^43^ thus initiating CD8^+^Tcell–mediated immune responses.^44^ Accordingly to a previous report,^45^ the increased number of tumors in IL‐37tg mice may be due to the CD8^+^ T deficiency in DMBA/TPA skin cancer. In our study, we discovered no difference in resting T cells between WT and IL‐37tg littermates (Figure S3A). We evaluated the infiltration of T cells in DMBA/TPA skin cancer in IL‐37tg mice and their WT littermates. We found that CD8^+^ T cell infiltration in the tumor microenvironment was reduced in the skin and SDLN of IL‐37tg mice compared to WT littermates (Figure 3A). Although the same result was obtained for CD4^+^ T cells in SDLN, no significant difference in this cell type was observed in the skin (Figure S3B). Furthermore, according to the negative control of skin and SDLN (Figure S3C), we examined the effector function of CD8^+^ T cells (expression of activation marker CD69, activation‐associated CD44, effector cytokine interferon (IFN)‐γ, lysosomal‐associated membrane protein‐1 in mouse skin and SDLN from DMBA/TPA‐treated WT and IL‐37tg. DMBA/TPA‐treated WT displayed a higher percentage of effector CD8^+^ T cells than that detected in IL‐37tg mice (Figure 3B). A reduction of CD4^+^ T cells and the CD4^+^ T cells expressing IFN‐γ (Figure S3D), contributed to the CD8^+^ CTL response.^46^ The primary target of thymic stromal lymphopoietin (TSLP) is myeloid DCs, and TSLP can induce DCs maturation and migration. Expression analysis through qPCR showed that IL‐37tg expressed a lower level of the stem cell marker TSLP and IFN‐γ in tumor‐bearing skin tissues and SDLN as compared with WT mice (Figure S3E,F). These results indicated that the IL‐37 transgene may impair the activation and function of CD8^+^ CTLs in DMBA/TPA skin cancer.

Many CD8^+^ T cells were rescued when DC migration was blocked by CCR7 neutralizing antibodies, along with the IFN‐γ^+^CD44^+^ cells and CD107a^+^CD44^+^ cells in CD8^+^ T cells (Figure S3G,H). These results indicated that CD8^+^ T cell infiltration and effector function was dependent on DC migration.

### 2.4 IL‐37 weakens the maturation and function of CD103^+^DCs

**FIGURE 4:**
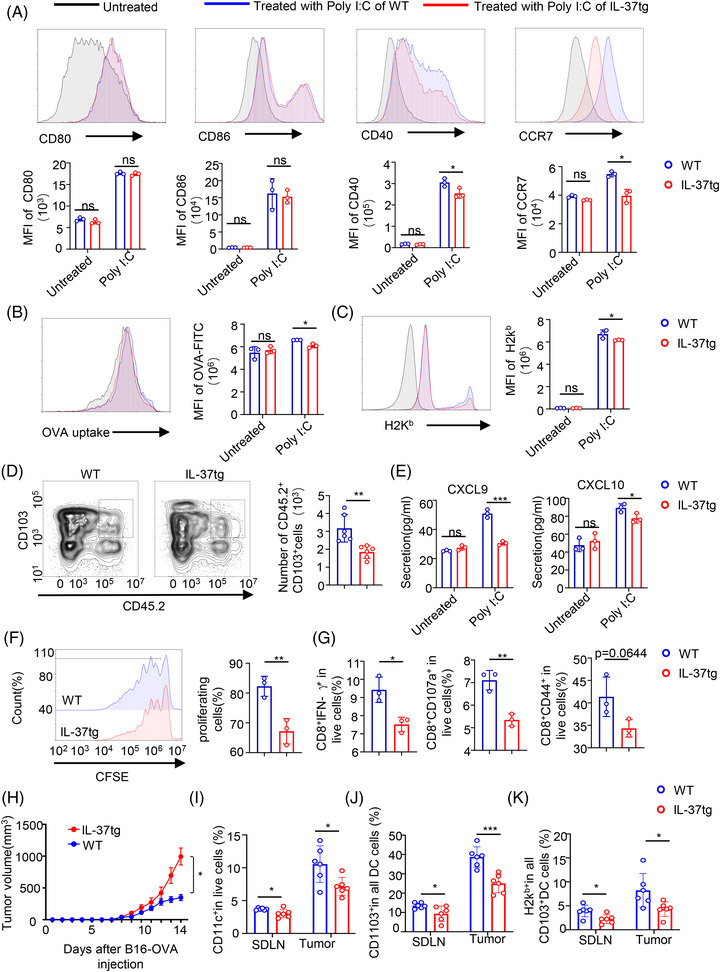
Interleukin (IL)‐37 inhibited the ability of CD103^+^DCs. CD103^+^DCs were isolated from the bone marrow of IL‐37b‐transgenic (IL‐37tg) mice and wild‐type (WT) littermates and induced with GM‐CSF and Flt3L. (A) Representative mean fluorescence intensity (MFI) (Upper) and histogram (Lower) of CD80, CD86, CD40, and CCR7 in CD103^+^DCs treated with or without 10 ng/ml poly(I:C) for 18 h from IL‐37tg mice and WT littermates. (B, C) 1 × 10^6^ Live CD103^+^DCs were incubated with 50 μg/ml ovalbumin (OVA)‐fluorescein isothiocyanate (FITC) protein for 45 min at 37°C after treatment with or without 10 ng/ml poly(I:C) for 18 h, representative histogram and MFI of OVA‐FITC and H2K^b^. (D) CD103^+^DCs that migrated to the skin‐draining lymph nodes (SDLNs) were sorted as CD11c^+^ CD45.2^+^ CD103^+^ live cells. (E) C‐X‐C motif chemokine ligand 9 (CXCL9) and CXCL10 secretion in the culture medium from isolated WT and IL‐37tg mouse CD103^+^DC treated with or without 10 ng/ml poly(I:C) for 18 h was measured by enzyme‐linked immunosorbent assay (ELISA), n = 3/group. (F) Isolated WT and IL‐37tg CD103^+^DCs were immunized with OVA protein for 1 h. Naive OT‐I CD8^+^ cells were isolated from spleens labeled with 5 μM CFSE and co‐cultured with CD103^+^DCs at a ratio of 5:1 pretreated with 10 ng/ml poly(I:C) for 18 and 72 h post‐coculture. Proliferation was measured by the CFSE dilution assay. Representative histograms of CFSE dilution (left) and statistical analysis of proliferating cells (right). (G) Quantification of FACS analysis of CD8^+^ CD107a^+^ cells, CD8^+^ IFN‐γ^+^ cells, and CD8^+^ CD44^+^ cells, n = 3/group. (H) B16‐OVA tumor growth curve of WT and IL‐37tg mice. n  =  6/group. (I‐J) The percentage of DC and CD103^+^DCs of SDLN and tumor. (K) The percentage of the H2K^b^ of CD103^+^DCs. All Data are presented as the mean  ±  SD *p  <  0.05; **p  <  0.01; ***p  <  0.001. ns, no significance, as determined by a two‐tailed Student's *t*‐test.

Given the results found in mice, we examined whether IL‐37 weakened the ability of CD103^+^DCs to prime CD8^+^ T cells in vitro, thus inhibiting the anti‐tumor immunity. The maturation, phagocytic activity, and migratory capacity of CD103^+^DCs were evaluated by using bone marrow‐derived CD103^+^DCs from either WT (WT‐CD103^+^DCs) or IL‐37tg mice (IL‐37tg‐CD103^+^DCs). CD103^+^DCs were then analyzed by flow cytometry and used in in vitro assays (Figure S4A). To examine the effect of IL‐37 on CD103^+^DCs maturation, we stimulated CD103^+^DCs with poly(I:C) in vitro and then analyzed their phenotype. After 18 h of poly(I:C) stimulation, a significant increase in the MFI of CD40 and CCR7 expression was observed in WT‐CD103^+^DCs compared to IL‐37tg‐CD103^+^DCs (Figure 4A). These findings were also observed in vivo (Figure 2D,E). To investigate whether IL‐37 affected the antigenic‐specific CD8^+^ T cell response of CD103^+^DCs, CD103^+^DCs were treated with poly(I:C) for 18 h and loaded with ovalbumin (OVA), and the capacity of CD103^+^DCs to uptake OVA‐fluorescein isothiocyanate (OVA‐FITC) was assessed. IL‐37tg‐CD103^+^DCs exhibited reduced phagocytic capacity of OVA (Figure 4B). In addition, IL‐37tg‐CD103^+^DCs showed a weakened cross‐presentation ability of the H2K^b^ molecules after OVA protein internalization compared with WT‐CD103^+^DCs (Figure 4C).

To determine whether IL‐37tg‐CD103^+^DCs displayed a weakened capacity of migration to the draining lymph nodes, an adoptive cell transfer experiment was performed, and we used poly(I:C) to pre‐treated CD45.2^+^CD103^+^DCs for 18 h, then CD45.2^+^CD103^+^DCs were injected subcutaneously into mice with background CD45.1. Then, 24 h post‐CD103^+^DCs injection, the number of DCs migrating from the adjacent SDLN as CD45.2^+^CD103^+^ among CD11c live cells was examined. CD45.2^+^CD103^+^DCs were analyzed using flow cytometry (Figure S4B). Notably, WT‐CD103^+^DCs had a stronger ability to migrate to the SDLN compared with IL‐37tg‐CD103^+^DCs (Figure 4D). To determine whether the inhibition of IL‐37 on CD103^+^DCs migration is mediated by CCR7, WT mice with CD45.1 background were pre‐treated with CCR7 neutralizing antibodies and IgG2A isotype. IL‐37tg‐CD45.2^+^CD103^+^DCs and WT‐CD45.2^+^CD103^+^DCs were adoptively transferred into mice with CD45.1 background and CD45.2^+^CD103^+^ DCs of SDLN were analyzed. The results displayed that there was no marked difference in the number of IL‐37tg‐CD45.2^+^CD103^+^DCs and WT‐CD45.2^+^CD103^+^DCs in SDLN of CD45.1 mice treated with CCR7 neutralizing antibody. Whereas the number of IL‐37tg‐CD45.2^+^CD103^+^DCs in SDLN treated with IgG2A was less than that of those WT‐CD45.2^+^CD103^+^DCs (Figure S4C). This indicated that the inhibition of IL‐37 on DCs migration is dependent on CCR7.

A significant reduction in the cytokines CXCL9 and CXCL10 released from IL‐37tg‐CD103^+^DCs as compared with those released from WT‐CD103^+^DCs was observed (Figure 4E). The ability to prime OT‐I CD8^+^T cells was also evaluated by incubating the CD103^+^DCs with CFSE‐labeled OT‐I CD8^+^T cells at a ratio of 1:5. After 72 h, the proliferation of OT‐I CD8^+^ T‐cells was analyzed. A significant decrease in OVA‐specific CD8^+^, CD8^+^ IFN‐γ^+^, CD8^+^CD107a^+^, and CD8^+^CD44^+^ OT‐I cells was observed in the IL‐37tg‐CD103^+^DCs compared with the WT‐CD103^+^DCs (Figure 4F,G). Taken together, these data indicate that IL‐37 weakens the maturation and function of CD103^+^DCs and inhibits CD8^+^ T cell sensitization. At the same time, to better understand the role of IL‐37 in anti‐tumor‐specific immunity, we established the B16‐OVA xenograft model in WT and IL‐37tg mice. The results displayed that the tumor growth of IL‐37tg mice was faster than that of WT littermates (Figure 4H). Then we analyzed the DCs in tumors and SDLN. The results showed that WT mice had more DC and CD103^+^DCs in tumors, and also presented H2K^b^ molecules compared with IL‐37tg mice (Figure 4I–K), which was consistent with the results found in the DMBA/TPA SCC model.

### 2.5 IL‐37 inhibits long‐time glycolysis of CD103^+^ DCs via SIGIRR

**FIGURE 5:**
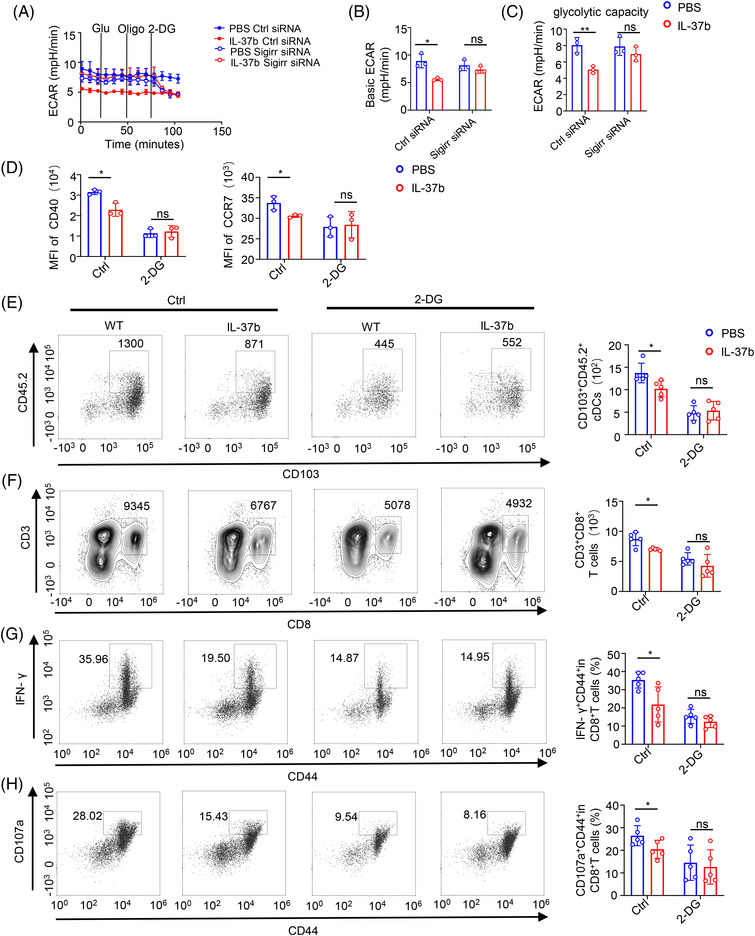
Glycolysis is required for dendritic cell (DC) function and migration. (A) Bone marrow‐derived CD103^+^DCs of mice were transfected for 72 h with the SMARTpool small‐interfering RNA (siRNA) reagent against single immunoglobulin interleukin‐1‐related receptor (SIGIRR) or with an Accell non‐targeting control siRNA. Analysis of the extracellular acidification rate (ECAR) of the transfected CD103^+^DCs stimulated in vitro with phosphate‐buffered saline (PBS), 100 ng/ml interleukin (IL)‐37b 2 h and treated with 10 ng/ml poly(I:C) for 18 h, in basal conditions and after sequential addition of glucose, oligomycin, and 2‐deoxyglucose (2‐DG) (*n* = 3/group). (B, C) Glycolysis and glycolytic capacity of CD103^+^DCs transfected with either SIGIRR‐siRNA or control‐siRNA (Ctrl). (D) Mean fluorescence intensity (MFI) of CD40 and CCR7 in CD103^+^DCs treated with or without 2‐DG at 5 mM. (E) CD103^+^DCs that migrated to the skin‐draining lymph node (SDLN) were sorted as CD11c^+^ CD45.2^+^ CD103^+^ live cells with or without 2‐DG‐treated. (F) The numbers of CD3^+^CD8^+^ T cells in SDLN with or without 2‐DG treatment. (G, H) The ratio of CD44^+^ IFN‐γ^+^ and CD44^+^ CD107a^+^in CD3^+^CD8^+^ T cells of SDLN with or without 2‐DG‐treated. Data are presented as the mean  ±  SD. Statistical significance was analyzed by a two‐tailed Student's *t*‐test. *p  <  0.05; **p  <  0.01.

Previous studies have shown that the trimer of IL‐37, SIGIRR, and IL‐18Rα, are essential for the function of IL‐37, and found that glycolysis can affect the migration of DCs.^47^, ^48^ Therefore, we hypothesized that IL‐37‐SIGIRR signaling might have a direct impact on CD103^+^DCs metabolism. SIGIRR was partially silenced in CD103^+^DCs using small‐interfering RNA (siRNA). Silencing efficiency was validated by qPCR and WB analysis (Figure S5A,B). Bone marrow‐derived CD103^+^DCs stimulated with poly(I:C) for 1 h with or without treatment with recombinant IL‐37 were evaluated using the Seahorse XFe24 Analyzer to detect the extracellular acidification rate (ECAR) and oxygen consumption rate (OCR). SIGIRR knockdown in CD103^+^DCs showed no ECAR and OCR change (Figure S5C,D). Nevertheless, after the CD103^+^DCs were pre‐treated for 18 h with poly(I:C), we found that recombinant IL‐37 treatment suppressed ECAR, and, more specifically, reduced glycolysis. Furthermore, the inhibitory role of recombinant IL‐37 was rescued by SIGIRR knockdown in CD103^+^DCs (Figure 5A–C), whereas no difference in OCR was observed (Figure S5E).

It has been reported that glycolysis can inhibit the function and migration of DCs. Therefore, the glycolytic inhibitor 2‐deoxyglucose (2‐DG) was added to CD103^+^ DCs. We found that 2‐DG can inhibit the expression of CD40 and CCR7 in CD103^+^ DCs, and this inhibition was dose‐dependent (Figure S5F). Moreover, 2‐DG can eliminate the difference between CD40 and CCR7 in CD103^+^ DCs after the treatment with or without recombinant IL‐37 (Figure 5D). In in vivo, the migration ability of 2‐DG treated CD103^+^ DCs decreased, and 2‐DG could abolish the difference in the migration number of CD103^+^ DCs and CD8^+^ T cells in WT and IL‐37tg mice (Figure 5E,F). Moreover, the difference in the percent of effector CD8^+^ T cells was also eliminated after 2‐DG treatment (Figure 5G,H). These results suggested that IL‐37 inhibited the maturation and function of CD103^+^ DCs via long‐term glycolysis. This is consistent with the results that were previously observed in IL‐37tg mice.

**FIGURE 6:**
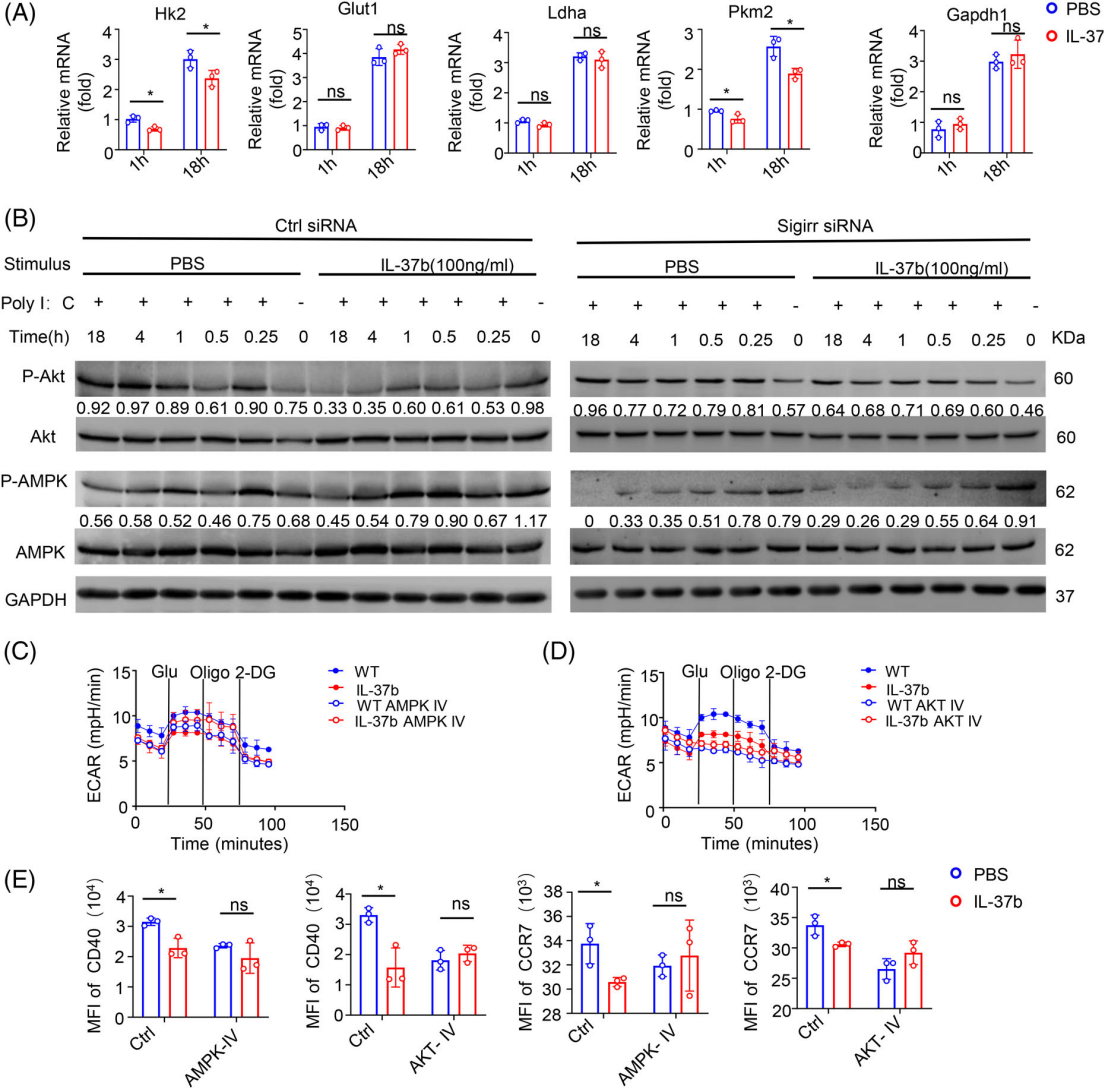
Interleukin (IL)‐37 limited long‐term glycolysis of CD103^+^DC cells via the single immunoglobulin IL‐1‐related receptor (SIGIRR) pathway. (A) Quantitative polymerase chain reaction (qPCR) analysis of gene expression of glucose transporter Glut1 and glycolytic enzymes Hk2, Ldha, Pkm2, and glyceraldehyde 3‐phosphate dehydrogenase (GAPDH) of CD103^+^DCs, transfected with control‐ small‐interfering RNA (siRNA) upon differential activation with poly(I:C) for 1 and 18 h, *n* = 3/group. (B) Immunoblotting was performed to detect the phosphorylation of Akt and activated protein kinase (AMPK) in Ctrl siRNA (left) and SIGIRR‐siRNA (right) transfected CD103^+^DCs. GAPDH was used as a loading control. (C, D) The extracellular acidification rate (ECAR) of the CD103^+^DCs treated with or without AMPK inhibitor dorsomorphin (Compound C) 2HCl at 10 μM treated for 30 min, the Akt inhibitor MK‐2206 2HCl at 5 μM treated for 30 min. (E) Mean fluorescence intensity (MFI) of CD40 and CCR7 in CD103^+^DCs treated with or without AMPK inhibitor Dorsomorphin (Compound C) 2HCl at 10 uM treated 30 min, Akt inhibitor MK‐2206 2HCl at 5 uM treated 30 min. All data are presented as mean  ±  SD. Statistical significance was analyzed by a two‐tailed Student's *t*‐test. *p  <  0.05; **p  <  0.01.

To estimate the effect of IL‐37 on the metabolic reprogramming of CD103^+^DCs, we examined the kinetics of the gene expression of key enzymes involved in glycolysis. Analysis through qPCR results showed that recombinant IL‐37 treatment slightly inhibited the expression of pyruvate kinase (Pkm2) and hexokinase (Hk2), whereas no effect on the expression of lactate dehydrogenase (Ldha), glyceraldehyde‐3‐phosphate dehydrogenase (GAPDH), and glucose transporter 1 (Glut1) was detected in the early stage of poly(I:C) stimulation of CD103^+^DCs (1 h after stimulation). At late time points (after 18 h stimulation), recombinant IL‐37 inhibited the expression of Pkm2, and Hk2, whereas no change in the expression of GAPDH, Glut1, and Ldha was observed (Figure 6A). The expression of glycolytic enzymes showed no significant difference after SIGIRR knockdown at both the early and late time points (Figure S6A).

The Akt pathway plays a key role in regulating the Toll‐like receptor (TLR)‐stimulated glycolytic metabolism of DCs.^31^ The analysis of the time process showed that treatment with recombinant protein IL‐37 showed a limited effect on Akt phosphorylation in the early stage of activation (within the first hour), whereas in the late stage after activation (about 4–18 h) the Akt phosphorylation in CD103^+^DCs treated with recombinant protein IL‐37 was gradually lost (Figure 6B, left). AMPK activation can block the stimulation of DCs glycolysis and metabolism induced by TLR. Although no change in AMPK phosphorylation in the late stage of activation was detected, IL‐37 treatment significantly increased AMPK phosphorylation in the early stage, which is consistent with a previous study reporting IL‐37‐mediated activation of AMPK.^49^ Interestingly, the IL‐37‐mediated inhibition of Akt phosphorylation in the late stage and the phosphorylation of AMPK in the early stage displayed a reverse trend when SIGIRR was knocked down (Figure 6B, right).

To determine whether the IL‐37‐SIGIRR signaling pathway inhibits glycolysis through AMPK‐ Akt signaling pathways, CD103^+^DCs were treated with AMPK and Akt inhibitors. CD103^+^DCs stimulated with poly(I:C) for 18 h with or without the treatment with recombinant IL‐37 were evaluated using the Seahorse XFe24 Analyzer to measure the ECAR. The results showed that ECAR inhibition of recombinant IL‐37 disappeared after the treatment with AMPK and Akt inhibitors (Figure 6C,D and Figure S6B–E), We also measured the content of lactic acid in CD103^+^DCs, and the results showed that there was no difference after the treatment with AMPK and Akt inhibitor (Figure S6F).

Moreover, we found that the CD40 and CCR7 of CD103^+^ DCs were rescued after the treatment with AMPK and Akt inhibitors (Figure 6E). These results suggested that the AMPK and Akt signaling pathways might be important in the maturation and migration of CD103^+^DCs. When CD103^+^DCs were treated with AMPK inhibitors, IL‐37 treatment cannot affect the Akt phosphorylation (about 4–18 h) (Figure S6G). Taken together, these results showed that IL‐37 could inhibit the long‐term glycolysis (about 4–18 h) of CD103^+^DCs through the SIGIRR‐AMPK‐Akt pathway, which was consistent with the difference in ECAR.

### 2.6 CD8^+^T cells was positively correlate with CD103^+^DCs in DMBA/TPA SCC

**FIGURE 7:**
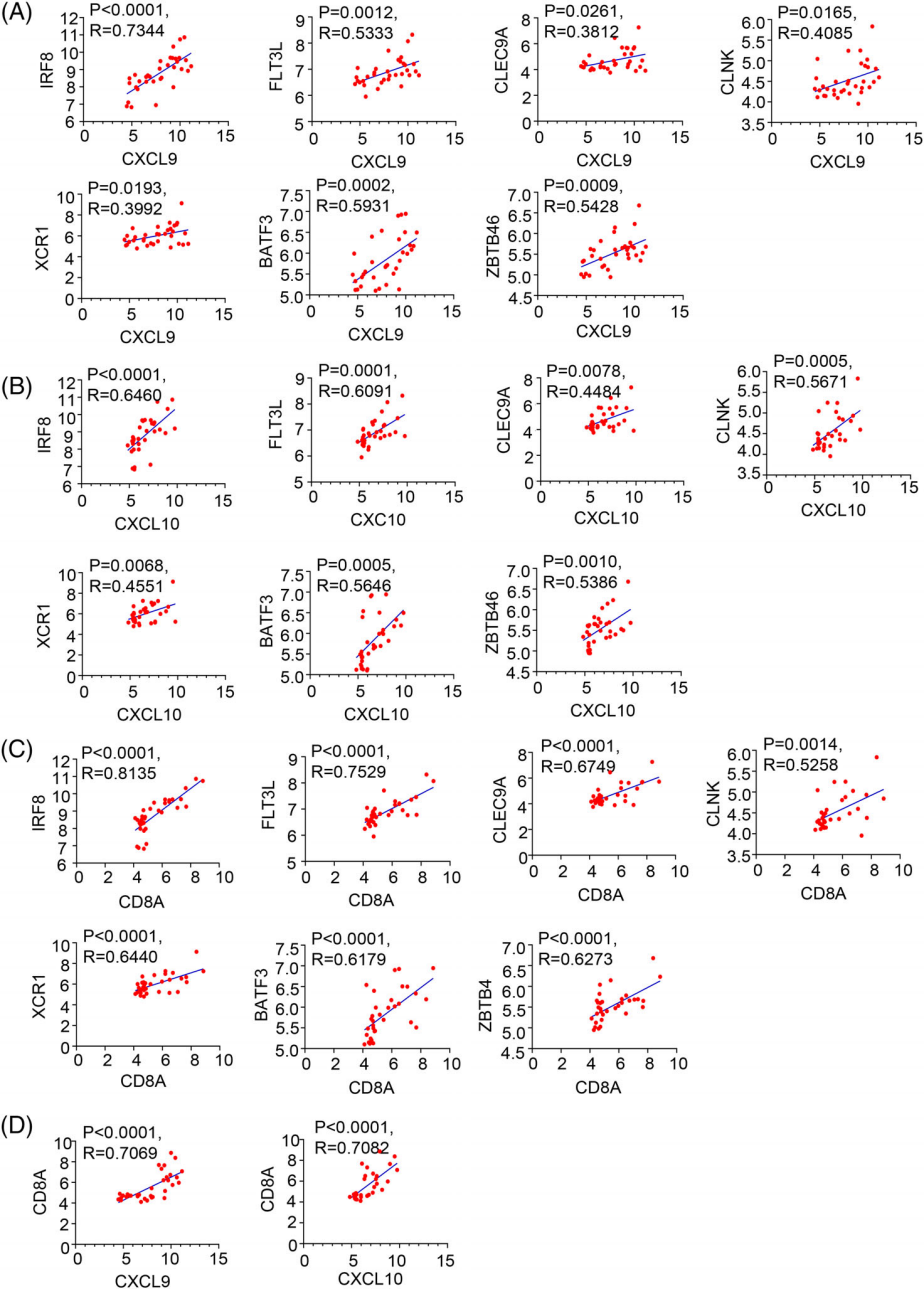
CD8^+^T cells positively correlated with CD103^+^DCs in mice with squamous cell carcinoma (SCC). (A–C) A significant correlation was found between the level of CD103^+^DC signature (IRF8, FLT3L, CLEC9A, CLNK, XCR1, BATF3, and ZBTB46) in 7,12‐dimethylbenz(a)anthracene (DMBA)/12‐O‐tetradecanoylphorbol‐13‐acetate (TPA) skin cancer mouse samples and chemokine C‐X‐C motif chemokine ligand 9 (CXCL9) (A), CXCL10 (B) and CD8A. (C) From the GEO database. Correlation is shown using R^2^ and significance was determined using a Spearman correlation. (D) A significant correlation was found between the level of CD8A in DMBA/TPA SCC (SCC, *n* = 33 mice) and chemokine CXCL9 and CXCL10.

**FIGURE 8:**
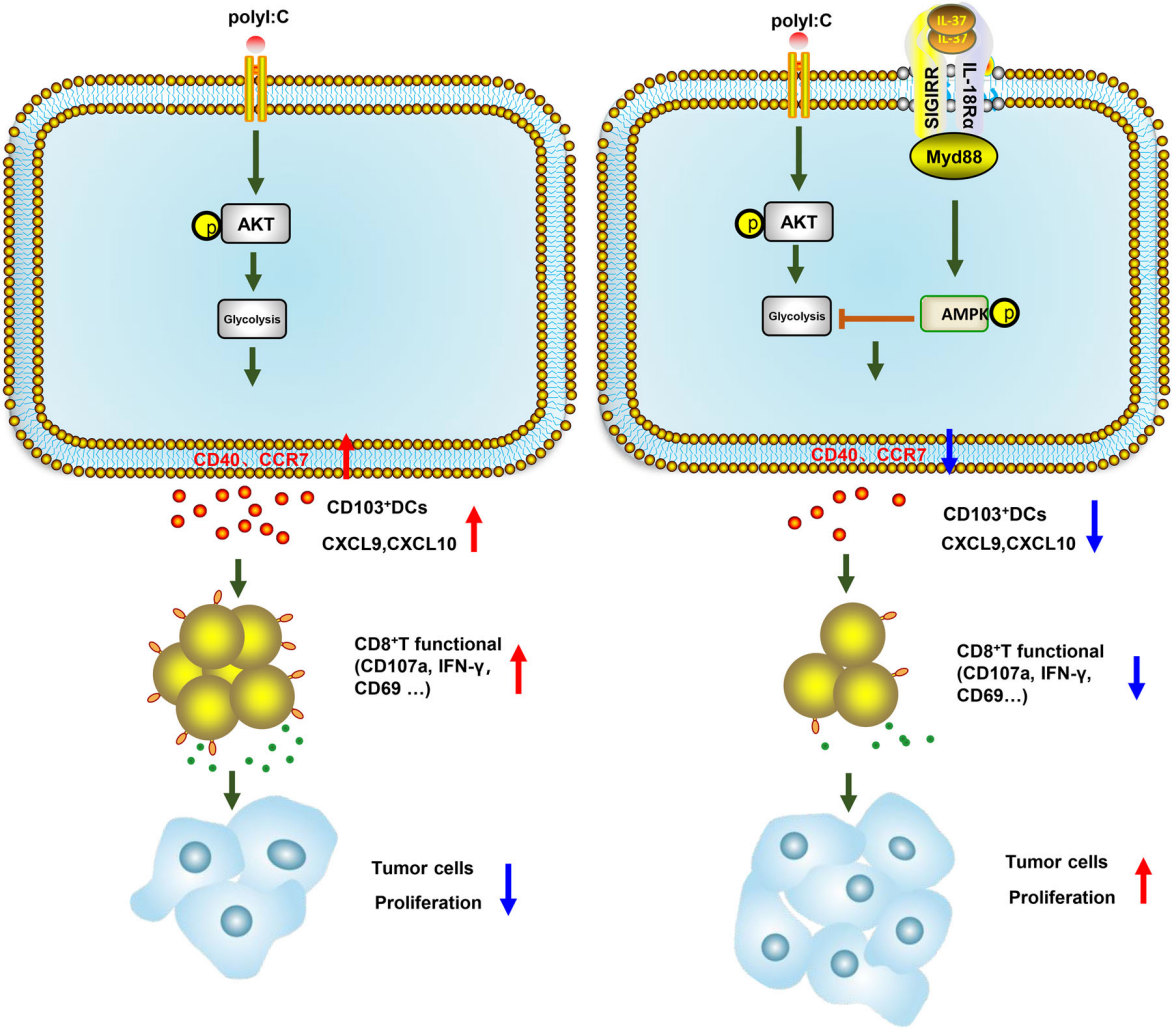
The mechanism of interleukin (IL)‐37 in 7,12‐dimethylbenz(a)anthracene (DMBA)/12‐O‐tetradecanoylphorbol‐13‐acetate (TPA) skin cancer. IL‐37 requires single immunoglobulin IL‐1‐related receptor (SIGIRR) to activate activated protein kinase (AMPK) and to inhibit mTOR/Akt signaling in CD103^+^DCs, subsequently leading to the suppression of CD8^+^ cytotoxic T lymphocytes (CTL) proliferation and function as well as the reduction in interferon (IFN)‐γ production. Dysfunctional CD8^+^ CTLs are deprived of their immune surveillance ability in tumors, thus enhancing tumorigenesis in skin cancer. The signaling pathway is designed with the help of Science Slides and Adobe Illustrator.

When analyzing GEO datasets for DMBA/TPA SCC (SCC, n = 33 mice), we observed a CD103^+^DCs signature, including IRF8, cytokine FLT3L, CLEC9A, CLNK, XCR1, BATF3, and ZBTB46,^50^, ^51^, ^52^, ^53^ which was positively correlated with CXCL9, CXCL10 and CD8A levels in DMBA/TPA SCC (Figure 7A–C). Moreover, CD8A showed a remarkable positive correlation with the chemokines CXCL9 and CXCL10 (Figure 7D). These findings enhanced the link between CD8^+^T cells and CD103^+^DCs in DMBA/TPA SCC and specifically clarified the mechanism underlying the role of IL‐37 in DMBA/TPA SCC (Figure 8).

## 3 DISCUSSION

The occurrence and development of tumors are heavily affected by the innate and adaptive immune system, which can either promote or weaken tumorigenesis.^53^, ^54^ Cytokines play a key role in regulating tumor immunogenicity and anti‐tumor immunity. Experimental studies have revealed the immune mechanism of IL‐1 family cytokines in melanoma, for example, IL‐18, IL‐33, IL‐36, and IL‐37.^21^, ^55^, ^56^, ^57^ However, the mechanism by which IL‐37 affects SCC, including its development, progression, and prognosis, remains poorly understood. Here, our study results support that IL‐37 weakens the function of CD103^+^DCs and affects the activation of CD8^+^ T cells to promote SCC development. We also showed that IL‐37 inhibited the metabolism of CD103^+^DCs via the SIGIRR‐AMPK‐Akt signaling pathway. It has been reported that IL‐1 family members play a greater role in skin cancer. In our study, we first examined the effect of IL‐37 on the immune microenvironment in DMBA/TPA‐induced mouse skin SCC.

Chronic inflammation promotes tumorigenesis.^58^ In the previous study, found that IL‐37 also played a role of anti‐inflammatory, inhibiting related inflammatory cells, including monocytes and monocyte‐derived DCs (MoDCs); however, tumor progression was also related to the loss of normal immune regulation processes. In the tumor microenvironment, cytotoxic T cells and DCs (especially the Batf3‐dependent CD103 subtype) or inflammatory factors produced by type I IFN, IFN‐γ, and IL‐12, are closely related to tumor prognosis.^59^, ^60^, ^61^, ^62^ Previous studies have reported that IL‐37 promotes CRC via cytotoxic T cells dysfunction.^21^ In our study, the results displayed that IL‐37 did not affect NK cells and Mø in tumor‐bearing skin tissues and SDLN, whereas a decrease in DCs was always detected in IL‐37tg mice, which was consistent with previous reports.^19^, ^63^ CD103^+^DCs play a major role in anti‐tumor immunity and have been proved to be related to the good prognosis of human cancer patients. They can secrete chemokines to recruit CD8^+^ T cells to initiate anti‐tumor immunity, making it an attractive potential immunotherapeutic target.^8^, ^13^, ^64^, ^65^ We further found that the reduction of DCs in IL‐37tg mice affected mainly the CD103^+^DCs, and a lower number of CD103^+^DCs in tumors associated with poor prognosis, which was consistent with the increased skin tumors in IL‐37tg mice. However, the effect of IL‐37 on CD103^+^DCs and its specific mechanism of action has not yet been reported. Further studies found that the expression of IL‐37 could inhibit the maturation (CD40) and migration (CCR7) of skin CD103^+^DCs. In addition, the level of CCR7 transcript in melanoma patients was related to the survival rate, indicating the potential role of cells expressing CCR7, including CD103^+^DCs, in anti‐tumor immunity.^66^ Given the activating effect of CD103^+^DCs on CD8^+^ T cells, we studied the composition of the T cells in mice and found that IL‐37tg mice had fewer CD8^+^ T cells than the WT littermates. To support the key role of IL‐37 in CD103^+^DCs, we induced bone marrow CD103^+^DCs in vitro using Flt3L and GM‐CSF.^44^, ^67^ We found that the MFI of CD40 and CCR7 in IL‐37tg‐CD103^+^DCs stimulated by poly(I:C) was lower than that detected in WT‐CD103^+^DCs, and the secretion of CXCL9 and CXCL10 was also lower, which was consistent with the results found in mice. To determine whether the ability of WT‐CD103^+^DCs to migrate to SDLN was enhanced, we performed adoptive cell transfer experiments. IL‐37 inhibited the migration of CD103^+^DCs. However, an important question is what are the molecular signals that inhibit CD103^+^DCs motility by IL‐37. Although it has been reported that IL‐37 inhibited MoDCs via glycolysis, its mechanism of action remains unclear. Glycolytic metabolism can affect the migration of DCs.^30^, ^34^ Molgora et al. reported that SIGIRR knockout resulted in NK cell‐mediated hepatoma resistance and defined SIGIRR as a functional checkpoint for NK cells.^17^ At the same time, in our previous studies, we also found that blocking SIGIRR could eliminate the inhibition of cytotoxic T‐cell effectors, and the formation of the IL‐37–SIGIRR–IL‐18Rα ternary complex was very important for the anti‐tumor immunosuppressive properties of IL‐37.^21^, ^47^ Our investigation showed that IL‐37 promoted the rapid phosphorylation of AMPK and inhibited the long‐time phosphorylation of Akt, and this trend was alleviated after SIGIRR knockdown. Akt is an important signaling pathway involved in glycolysis.^68^, ^69^, ^70^ Consistently with previous studies, we also found that IL‐37 activated AMPK and inhibited the Akt signaling pathway, whereas SIGIRR depletion blocked IL‐37‐mediated activation of AMPK and inhibited TLR‐dependent glycolysis thus antagonizing TLR‐induced DCs maturation.^31^, ^33^, ^49^

CXCL9 and CXCL10 were mainly secreted by CD103^+^DCs,^8^ and also secreted by M1 Mø and Th1 cells. We found a consistent decrease in CXCL9/CXCL10 and CD103^+^DCs. Similar results were also obtained from the GEO database of the mouse DMBA/TPA model. Moreover, CD103^+^DCs‐related chemokines were positively correlated with CD8^+^ T cell markers in mouse SCC. The increase in tumor‐infiltrating CD103^+^DCs was reported to be closely associated with the anti‐tumor effects in different kinds of cancer, including metastatic melanoma, breast cancer, and lung adenocarcinoma.^64^, ^71^, ^72^ Our research results suggest that the mechanism by which IL‐37 acts on CD103^+^DCs may represent a potential target for the clinical development of anti‐tumor drugs for patients with SCC.

In conclusion, our finding identified a novel regulatory mechanism of IL‐37 in tumor immunity. In addition, our data strengthen the understanding of IL‐37 and emphasizes the important role of IL‐37 in cancer. Our findings on the interaction of IL‐37 and CD103^+^DCs in the tumor microenvironment will help to develop new strategies for cancer treatment strategies.

## 4 MATERIALS AND METHODS

### 4.1 Animals

IL‐37tg mice, homozygous for IL‐37b, came into being with a C57BL/6 background, as previously suggested.^21^ WT mice were used as littermates for the IL‐37tg mice. OT‐I mice on a C57BL/6 background have also been previously described.^21^ CD45.1 mice on a C57BL/6 background were fed and maintained in our laboratory. All mice were male mice in the experiment in this article. All the mice were raised in a particularly pathogen‐free animal facility at Sichuan University.

### 4.2 DMBA/TPA skin cancer model

For DMBA/ TPA‐induced skin tumors, the back of 6–8‐week‐old mice were shaved and applied with a bolus dose of 50 μg DMBA in 200 μl acetone (Sigma) for one week, after that twice a week applications with 5 μg TPA in 200 μl acetone (Sigma) for 32 weeks.^73^ The number and size of the tumors on the backs of the mice were recorded weekly. The experiment was completed after 32 weeks of TPA treatment. Biopsies of both tumors and nontumorigenic dorsal skin were gathered and applied for either histology, flow cytometry analysis, or RNA isolation.

### 4.3 B16‐OVA tumor model

For the B16‐OVA tumor model, IL‐37tg mice and WT littermates were subcutaneously injected on the flank with 5 × 10^5^ B16‐OVA cells. Tumors were measured by the same vernier caliper every day, the volumes were calculated, and then tumors were collected for flow analysis.

### 4.4 Skin cell preparation

To isolate the skin cells, the back skin tissue was removed and, cut into small pieces, after scraping off the subcutaneous fat with a razor blade. The skin samples were incubated with collagenase IV (Sigma), a concentration is 1 mg/ml, and DNase I (Sigma), a concentration is 0.1 mg/ml for 90 min at 37°C on a shaker with vigorous shaking. The skin suspension was filtered with a 70‐μm filter, and the erythrocytes in the filtered skin suspension were lysed in an erythrocyte lysis buffer (Solarbio). Individual cells were analyzed using flow cytometry.

### 4.5 Flow cytometry

Cells were obtained from the mouse lymph nodes and skin tissue, digested with collagenase, and filtered to obtain single‐cell suspensions. Isolated cells were stained for cell membranes and then intracellularly in the presence of brefeldin A (BD Pharmingen) after re‐stimulation with phorbol myristate acetate/ionomycin (Sigma) for 4 h. Intracellular staining was performed based on a previously described protocol.^21^ The flow antibodies used for flow‐through assays were obtained from BioLegend or BD. NovoExpress flow cytometry was used to collect flow cytometry data and use NovoExpress software (ACEA Biosciences) was for analysis.

### 4.6 CCR7 intervention

To block the migration of CD103^+^DCs in mice, WT and IL‐37tg mice that were treated with DMBA/TPA were intravenously injected with monoclonal rat anti‐mouse CCR7 neutralizing antibody (R&D Systems) (0.5 μg/10 g /dose) every day for 20 weeks. Then, the tumor tissues and SDLN were harvested and analyzed by flow cytometry.^74^

To detect the inhibition of IL‐37 in CD103^+^DC migration is mediated by CCR7, WT mice with a background of CD45.1 were treated with CCR7 neutralizing antibody (0.5 μg/10 g /dose) (R&D Systems) and IgG2A isotype (R&D Systems) until the end of the experiment.

### 4.7 Generation of bone marrow‐derived CD103^+^DCs

CD103^+^ DCs were induced as previously reported by Mayer *et al*.^44^ Bone marrow cells were gathered from the tibia and femur of the mice. Cells were resuspended at 1 × 10^6^ bone marrow cells per 10 cm dish in complete RPMI‐1640 (Gibco) containing 10% fetal bovine serum (FBS, Excell), 1% antibiotic–antimycotic (Sigma), 50 μM β‐mercaptoethanol (Sigma), 20 ng/ml recombinant murine‐granulocyte–macrophage colony‐stimulating factor (GM‐CSF) (PeproTech), and 100 ng/ml recombinant murine Flt3L (PeproTech). On day 3, after the plate was rotated slightly, remove the nonadhesive cells and collect them by centrifugation, and replace the solution with 5 ml of fresh culture medium. The purity of the isolated cells was analyzed by flow cytometry using anti‐mouse CD11c antibody until harvest on the 15th to 16th day of culture.

### 4.8 CD103^+^DCs stimulation and migration assay in vivo

CD103^+^DCs were plated in low adhesion six‐well plates in RPMI‐1640 (Gibco) with 10% FBS (Excell), and 1% antibiotic–antimycotic(sigma) and were either untreated or treated with poly(I:C) (APExBIO) for 18 h. The samples were digested with trypsin without ethylenediaminetetraacetic acid, and the flow cytometry antibody was used to analyze the surface marker. For the CD103^+^DC migration assay, CD103^+^DC were washed with sterile phosphate‐buffered saline (PBS) according to the manufacturer's protocol, resuspended in 200 μl, and then injected subcutaneously in the flank side of CD45.1 mice. Three days after CD103^+^DCs injection, SDLNs were collected and used for single‐cell isolation. The single cells were then analyzed by flow cytometry.

To investigate the effects of AMPK, Akt, and 2‐DG of CD40 and CCR7 on CD103^+^DCs and CD103^+^DCs were treated with 10 μM of AMPK inhibitor Dorsomorphin (Compound C)2HCl (Selleck) for 30 min, 5 μM of Akt inhibitor MK‐2206 2HCl (Selleck) for 30 min and 1 mM, 2 mM, 5 mM 10 mM of 2‐DG(Adamas) before treating with poly(I:C).

In order to study the effect of 2‐DG on the activation and migration of CD103^+^DCs, the mice were intraperitoneally injected with 500 mg/kg 2‐DG in a volume of 200 μl or 200 μl control PBS. CD103^+^DCs with poly(I:C) was injected in the right footpad of CD45.1 mice. Three days after CD103^+^DCs injection, SDLNs were collected and used for single‐cell isolation. The single cells were then analyzed by flow cytometry.

### 4.9 Antigen uptake and peptide presentation assay

Chicken OVA labeled with Alexa Fluor 488 (AF488‐OVA) was used (Thermo Fisher) to detect the antigen uptake and endocytosis of bone marrow‐derived CD103^+^DCs of WT and IL‐37tg. Briefly, WT and IL‐37tg CD103^+^DCs were treated with poly(I:C) for 18 h. The control was not given any treatment. CD103^+^DCs were then incubated with AF488‐OVA (50ug/ml) at 37°C for 45 min, washed, and dead cells were labeled with 7‐AAD viability staining solution (BioLegend). The MFI of OVA‐AF488 in living cells was measured by flow cytometry. To measure the ability of OVA peptide presentation to MHC‐I, CD103^+^ DCs were treated with poly(I:C) for 18 h and incubated with OVA protein (50 ug/ml) at 37°C for 1 h. The cells were then washed with PBS, and stained with H2K^b^‐APC (Biolegend) for about 15 min at room temperature. CD103^+^DCs without OVA treatment and isotype controls were used as negative control and staining controls separately. H2K^b^ levels were measured by flow cytometry.

### 4.10 T‐cell isolation

The mouse spleen was removed and pressed on a 40 μm filter. The filtered cells were washed with sterile PBS and centrifuged. Naive CD8^+^ T cells were purified from centrifuged cells according to the manufacturer's protocol, and the Mouse Naive CD8^+^ T Cell Isolation Kit was obtained from STEMCELL, FACS analysis revealed that the purity of T cells was > 90%.

### 4.11 Seahorse assay

BM‐derived CD103^+^DCs were treated with 100 ng/ml IL‐37b (R&D) for 2 h, followed by stimulation with poly(I:C) (10 ng/ml) for 1 h and 18 h. Real‐time changes in OCR and ECAR were analyzed using a Seahorse XFe24 analyzer. CD103^+^DCs were inoculated in XF‐24 cell culture plates with 1.5 × 10^5^ cells/well in RPMI 1640 medium. After incubation at the indicated time points, the medium was replaced with warm Seahorse assay medium (XF assay basal medium containing 10 mM glucose, unless specified without glucose, 10% FCS, and 2 mM l‐glutamine, pH 7.4), and the assay medium was added to bring the final volume to 500 μl/well. For glycolytic stress testing following the ECAR baseline measurements, glucose, oligomycin A, and 2‐DG were added sequentially to each well to reach 10 mM, 1 μM, and 50 mM, respectively. For OCR evaluation, oligomycin, carbonyl cyanide 4‐(trifluoromethoxy) phenylhydrazone, and rotenone/antimycin A were added sequentially to each well to reach final concentrations of 1.5, 1.0, and 0.5 μM, respectively.

### 4.12 siRNA‐mediated silencing of gene expression

CD103^+^DCs were cultured as described previously. siRNA oligonucleotide duplexes (Dharmacon)^17^ were transfected with Accell Mouse SIGIRR SMART pool siRNA to block SIGIRR expression. SIGIRR siRNA reference sequences were as follows: CCUACGUGUCCUAUAGCGA, CCCUGCUCUAUGUUAAGUG, UCGUGGUUCUUUCAGAUGC, and GGAUGAUGUGUAGCCCAUA. The absence of complementary RNA sequences in the targeted genome (Dharmacon) was used as a control. After 72 h of incubation, the delivery medium was replaced with RPMI 1640 medium.

### 4.13 Western blotting

The collected cells were lysed and resolved by 12% sodium dodecylsulfate–polyacrylamide gel electrophoresis and transferred to a polyvinylidene fluoride membrane (MerckMinipore). The blots were probed with primary antibodies. Primary antibodies were obtained from Cell Signaling Technology and included antibodies against the following targets, Akt, p‐Akt S473, AMPKα, phosphor (p)‐AMPKα, and GAPDH, and SIGIRR were acquired from Abcam. The primary antibodies were detected using horseradish peroxidase‐conjugated goat anti‐rabbit antibodies (Invitrogen, A27036 1:10000 dilution) and further visualized using ECL reagents (Merck Millipore, WBKLS0500). ImageJ software was used to further quantify the band intensity in the image, including only the band intensity in the linear range.

### 4.14 Hematoxylin and eosin staining

Mouse skin tumors were fixed with 4% paraformaldehyde, embedded in paraffin according to the laboratory operation guide, then cut into 5 μm sections, which were stained with hematoxylin and eosin and examined via histopathological analysis. Pictures were acquired by using an Olympus BX600 microscope and a SPOT Flex camera.

### 4.15 RNA isolation and qRT‐PCR

Total tissue and cellular RNA were isolated using TRIZOL (Life Technologies). DNA was removed, and PrimeScript RT kits and gDNA Erase (Takara) were used for cDNA synthesizing. Premix Ex Taq II was regarded to determine the relative gene expression. Gene expression was quantified using the primers listed in Table S1. GAPDH and hypoxanthine phosphoribosyltransferase was used as endogenous control, and mRNA expression was calculated by the ΔΔCt method to evaluate mRNA expression ploidy changes. All ploidy changes were normalized to those of the untreated control.

### 4.16 Enzyme‐linked immunosorbent assay

The medium and serum of SCC mice were saved and examined the chemokine content using a CXCL9 enzyme‐linked immunosorbent assay (ELISA) kit (Sigma) and CXCL10 ELISA kit (Neo Bioscience), according to the user's operation process.

### 4.17 Statistical analysis

GraphPad Prism 8.0 software was used for the statistical analysis of the data. Tumor incidence was analyzed using the log‐rank 2 test to compare the curves of mice with tumors. Tumor diversity was analyzed using two‐way repeated measures analysis of variance (ANOVA). The malignant transformation rates and the percentage of mice with malignant tumors were analyzed using Fisher's exact test. The Mann‐Whitney test was used to compare data from two groups; ANOVA tests were used to compare three or more groups *p < 0.05, **p < 0.01, and ***p < 0.001.

## AUTHOR CONTRIBUTIONS

Jiong Li and Fan‐lian Zeng conceptualized the study; Jiong Li, Fanlian Zeng, and Zhen Wang designed the methodology, and Fan‐lian Zeng, Xiao‐yan Wang, and Ya‐wen Hu performed the experiments. Zhen Wang, Ya Li, Jing Hu, Jia‐dong Yu, Pei‐Zhou, Xiu Teng, Hong Zhou, Fu‐lei Zhao, Cheng‐cheng Yue, Hua‐ping Zheng, Shu‐wen Chen, Juan Cheng, Yan Hao, Qi‐xiang Zhao, Chen Zhang, Lin‐na Gu, Guo‐lin Li, Song Zou, Nongyu Huang, Zhong‐lan Hu, Wenlin Wu, Xiao‐qiong Wei, Xiao Liu, Yifan Zhou, Kaijun Cui, and Wei Li provided key reagents. Fanlian Zeng, Xiaoyan Wang, Yawen Hu, Zhen Wang, and Jiong Li wrote and revised the manuscript. All authors have read and agree with the final manuscript.

## CONFLICT OF INTEREST STATEMENT

The authors declare no conflict of interest.

## ETHICS STATEMENT

The operation process of experimental was approved by the Animal Experiment Ethics Committee from Sichuan University. The experimental procedure was used in accordance with the guidelines for the care and use of laboratory animals (NIH Publication No. 85‐23).

## Supporting information

Supporting Information

## Data Availability

Quantitative data supporting this study can be obtained in this paper and supplements. At the reasonable request of the magazine, the corresponding author of this article can provide all other data supporting the research results. RNA‐Seq data are available in Gene Expression Omnibus (GEO) database GSE63967 (https://www.ncbi.nlm.nih.gov/geo/geo2r/?acc = GSE63967).
