# Supplementary material for: Interleukin‐37 promotes DMBA/TPA skin cancer through SIGIRR‐mediated inhibition of glycolysis in CD103+DC cells
Source: MedComm (2020). 2023 Mar 5;4(2):e229. doi: 10.1002/mco2.229 (PMC9986080; doi:10.1002/mco2.229)
Supplement: Supplementary file 1 — Supporting Information [file MCO2-4-e229-s001.docx]

**Supplemental Information**

**Interleukin-37 promotes DMBA/TPA skin cancer through SIGIRR-mediated inhibition of glycolysis in CD103^+^DC cells**

Fan-lian Zeng,Xiao-yan Wang,Ya-wen Hu, Zhen Wang,Ya Li,Jing Hu, Jia-dong Yu, Pei-Zhou, Xiu Teng, Hong Zhou, Hua-ping Zheng, Fu-lei Zhao, Lin-na Gu, Cheng-cheng Yue, Shu-wen Chen, Juan Cheng, Yan Hao,Qi-xiang Zhao, Chen Zhang, Song Zou, Zhong-lan Hu, Xiao-qiong Wei, Xiao Liu, Guo-lin Li,Nong-yu Huang,Wen-ling Wu, Yi-fan Zhou, Wei Li, Kaijun Cui and Jiong Li

**Supplemental Figures**

**Figure S1:** (A) Representative H &E image of the skin at 8 weeks of IL-37tg mice and WT mice. (B) The epidermal thinness(μl) in IL-37tg mice and WT littermate, n = 7/group. (C) Representative immunoblots of the IL-37 in IL-37tg mice and WT mice. (D) RT-qPCR analysis was performed for IL-37 gene from IL-37tg mice and WT mice different tissues, n=5/group. Data are presented as mean ± SD. *P < 0.05; **P < 0.01; ***P < 0.001; ns, not significant, as determined by the two-tailed Student’s t-test.

**Figure S2:** (A) The numbers of CD11C^+^MHC II^+^ cells (DC cells), CD11B^+^F4/80^+^ cells (Mø cells), NK1.1^+^ cells (NK cells), and LCs (CD11C^+^ CD207^+^EpCAM^+^) in the skin tissue and (B) SDLN in IL-37tg mice and WT littermate mice untreated with DMBA/TPA, n = 6/group. (C) The number of CD11b^+^DC in the tumor-bearing skin tissue and SDLN (n = 6/group). (D-E) Q-PCR analysis of B220, BST2 and Siglec-H from the tumor-bearing skin tissue and SDLN of IL-37tg and WT mice, n=6/group. (F) gating strategy of Flow cytometry for the identification of cDCs (CD103^+^cDCs and CD11b^+^cDCs), macrophages, monocytes and moDDCs on week 6 from IL-37tg and its littermates mice treated with DMBA/TPA, n = 6/group. (G-H) The numbers of macrophages, monocytes, moDDCs , CD103^+^cDCs and CD11b^+^cDCs in the skin tissue (G)and SDLN (H). (I) Q-PCR analysis of IL-10, IL-12, and IL-2 from the tumor-bearing skin tissue and SDLN of IL-37tg and WT mice, n=6/group. Data are presented as mean ± SD. *P < 0.05; **P < 0.01; ns, not significant, as determined by the two-tailed Student’s t-test.

**Figure S3:** (A) Number of T cells in skin tissue and SDLN in IL-37tg mice and WT littermate mice untreated with DMBA/TPA, n = 6/group. (B) CD4^+^ cells in the skin tissue and SDLN in IL-37tg mice and WT littermate mice in the resting state, n=6/group. (C) The isotype control of Figure 3. (D) The ratio of CD4^+^ IFN-γ^+^ cells in the skin tissue and SDLN in IL-37tg mice and WT littermate mice in the resting state, n=6/group. (E-F) Q-PCR analysis of TSLP and IFN-γ in tumor-bearing skin tissue (E) and SDLN(F) of IL-37tg mice and WT mice, n=6/group. (G-H) Number of CD8^+^T cells and the ratio of IFN-γ^+^ CD44^+^ cells and CD107a^+^ CD44^+^ cells in CD8^+^T cells in SDLN of IL-37tg mice and WT littermate mice treated with CCR7 neutralizing antibodies, n = 6/group. Data are presented as mean ± SD.(H) *P < 0.05; **P < 0.01; ns, not significant, as determined by the two-tailed Student’s t-test.

**
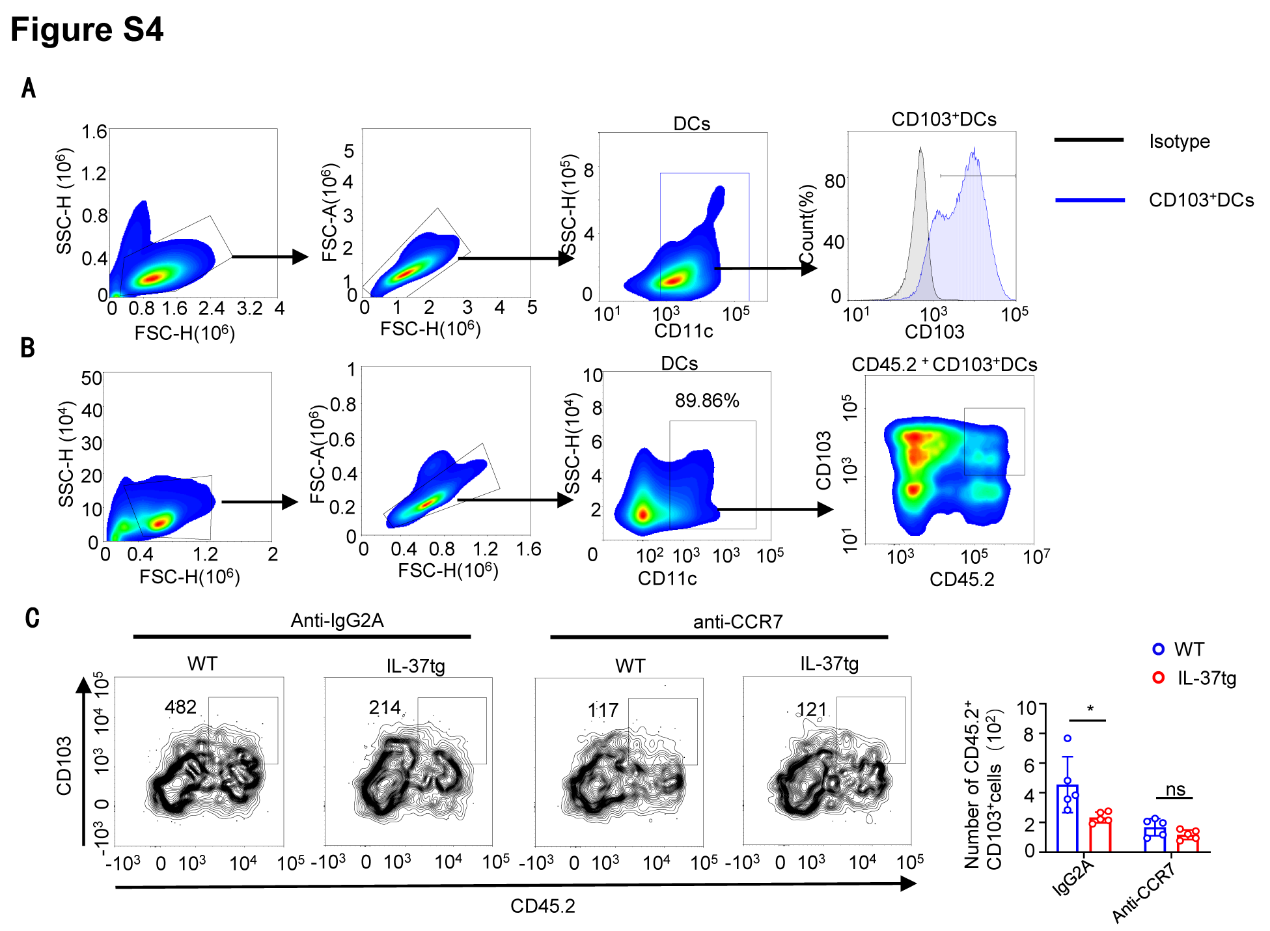
**

**Figure S4: Gating strategy used to identify the CD103^+^DC cells by flow cytometry.** (A) The ratio of bone marrow-derived CD103^+^DC. (B) Gating of CD11c^+^CD45.2^+^CD103^+^DC cells in the SDLN. (C) CD103^+^DCs that migrated to the SDLN were sorted as CD11c^+^ CD45.2^+^ CD103^+^ live cells after treated with CCR7 neutralizing antibody and IgG2A.

**Figure S5: Bone marrow-derived CD103^+^DC cells were transfected for 72 h with either the SMARTpool siRNA-SIGIRR or Accell nontargeting control siRNA.** (A) Q-PCR analysis of SIGIRR in CD103^+^DC. (B) Western Blot analysis of SIGIRR in CD103^+^DC. (C) Analysis of ECAR of the transfected CD103^+^DC cells stimulated *in vitro* with PBS and 100 ng/mL IL-37b 2h and then treated with 10 ng/mL poly(I:C) for 1 h, under basal conditions and after sequential addition of glucose, oligomycin, and 2-DG (n=3/group). (D) Analysis of OCR of the transfected CD103^+^DCs stimulated *in vitro* with PBS and 100 ng/mL IL-37b for 2h and then treated with 10 ng/mL poly(I:C) for 1 h under basal conditions and maximal conditions after sequential addition of oligomycin, FCCP, and rotenone with antimycin A (n=3/group). (E) OCR analysis of transfected CD103^+^DC cells stimulated *in vitro* with PBS and 100 ng/mL IL-37b for 2h before treated with 10 ng/mL poly(I:C) for 18 h, under basal conditions and maximal conditions after sequential addition of oligomycin, FCCP, and rotenone with antimycin A (n=3/group). (F) Poly(I:C)-induced activation of CD103^+^DCs in the presence of the glycolytic inhibitor 2-DG. Flow cytometry was used to measure surface expression of CD40 and CCR7 after poly(I:C) stimulation in the presence of the indicated concentrations of 2-DG. Unstimulated CD103^+^DCs cultured in the absence of 2-DG or poly(I:C) are shown for comparison. All data are presented as mean ± SD. Statistical significance was analyzed by Two-tailed Student’s T-test. *P < 0.05; **P < 0.01.

**Figure S6:** (A) Q-PCR analysis of gene expression of glucose transporter Glut1 and glycolytic enzymes Hk2, Ldha, Pkm2, and Gapdh1 in SIGIRR siRNA-transfected CD103^+^DC cells were measured after differential activation with poly(I:C) for 1 h and 18 h. n=3/group. (B-E) Glycolysis and glycolytic capacity of CD103^+^DCs treated with AMPK inhibitor Dorsomorphin (Compound C) 2HCl at 10 uM treated for 30 mins. AKT inhibitor MK-2206 2HCl at 5 uM treated for 30 mins. (F) Glycolytic metabolites of lactic acid in CD103^+^DCs after treatment with AMPK inhibitor Dorsomorphin (Compound C) 2HCl at 10 uM treated for 30 mins. AKT inhibitor MK-2206 2HCl at 5 uM treated for 30 mins. (G) Immunoblotting was performed to detect phosphorylation of AKT of CD103^+^DCs treated with AMPK inhibitor, GAPDH was used as a loading control. All data are presented as mean ± SD. Statistical significance was analyzed by Two-tailed Student’s T-test. ns, not significant.

**Table S1. Primer nucleotide sequences for qRT-PCR**

| **Gene** | | **5' primer** | | **3' primer** |
| --- | --- | --- | --- | --- |
| IL-10  IL-12  IL-2  TSLP  IFN- γ  CXCL9  CXCL10  Gapdh  Hk2  Glut1  Ldha  HPRT  SIGIRR  ACTB | TAACTGCACCCACTTCCCAG  ACTCTGCGCCAGAAACCTC  GCCCCAAGGGCTCAAAAATG  ACGGATGGGGCTAACTTACAA  TCAAGTGGCATAGATGTGGAA  ATCTTCCTGGAGCAGTGTGG  GTGAGAATGAGGGCCATAGG  GTCGGTGTGAACGGATTTG  CCGTGGTGGACAAGATAAGAGAG  CTGGACCTCAAACTTCATTGTGG  TGTCTCCAGCAAAGACTACTGT  GTTGGGCTTACCTCACTGCT  CTGCCGCTGGGTCTGTC  CCTCTATGCCAACACAGTGC | | AAGGCTTGGCAACCCAAGTA  CACCCTGTTGATGGTCACGAC  GCGCTTACTTTGTGCTGTCC  AGTCCTCGATTTGCTCGAACT  TGGCTCTGCAGGATTTTCATG  AGTCCGGATCTAGGCAGGTT;  GGCTAAACGCTTTCATTAAATT  TAGACTCCACGACATACTCAGC  GGACACGTCACATTTCGGAGC  GGGTGTCTTGTCACTTTGGCTG  GACTGTACTTGACAATGTTGGG  TAATCACGACGCTGGGACTG  TGGCTCTTCTGGGACCTCTT  ACATCTGCTGGAAGGTGGAC | |
